# Cognitive and Emotional Determinants of Automatic Perspective Taking in Healthy Adults

**DOI:** 10.3389/fpsyg.2022.883929

**Published:** 2022-05-02

**Authors:** Cristelle Rodriguez, Marie-Louise Montandon, François R. Herrmann, Alan J. Pegna, Panteleimon Giannakopoulos

**Affiliations:** ^1^Division of Institutional Measures, Medical Direction, Geneva University Hospitals, Geneva, Switzerland; ^2^Department of Psychiatry, Faculty of Medicine, University of Geneva, Geneva, Switzerland; ^3^Department of Rehabilitation and Geriatrics, Geneva University Hospitals and University of Geneva, Geneva, Switzerland; ^4^School of Psychology, University of Queensland, Brisbane, QLD, Australia

**Keywords:** altercentric interference, attention, automatic perspective task, theory of mind, social cognition

## Abstract

Previous studies using the dot-perspective task postulated that people automatically take into account others' perspective even when it prevents them from achieving their own goals. This human ability may be of key importance for the ascription of mental states and social interactions. The cognitive and emotional determinants of automatic perspective taking (APT) is still matter of debate. To address this issue, we examined the performance in the Samson et al. APT task in 91 healthy adults who underwent a detailed neuropsychological testing including assessment of their general intelligence (Wechsler Adult Intelligence Scale, WAIS), attention and impulsivity (Conners' Continuous Performance Test-II, CPT-II), alexithymia (Toronto Alexithymia Scale, TAS), and measures of affective empathy and explicit theory of mind (Geneva Social Cognition Scale, GeSoCS, and mini-Social cognition and Emotional Assessment, mini-SEA). Univariate and multiple linear regression models (adjusted for age, gender, and education) were used to explore the association between mean reaction times (respectively, mean number of errors) in the APT task, and the CPT-II parameters, WAIS global score (as well as subscale scores), TAS, and GeSoCS and mini-SEA scores. Only the CPT-II parameters were significantly associated with the mean reaction times. Increased omissions, commissions, and detectability as well as hit reaction time standard error in CPT-II were all related to worse performances both in Self and Other conditions. The mean number of errors was negatively associated with the GeSoCS score. Among the variables studied, only CPT-II parameters had a significant impact on egocentric and altercentric interference. Neither global intelligence nor alexithymia have an effect on dot-perspective task performance. The present findings suggest that people with lower attentional resources and increased impulsivity display worse performances in the APT task and are less responsive to both egocentric and altercentric interference.

## Introduction

Empathy is a complex construct that determines our abilities for social interaction. It includes an affective component (affective empathy) that refers to the capacity of sharing emotions and respond to the emotions of others and a cognitive component (cognitive empathy) that partly overlaps with the concept of the theory of mind (ToM), namely, the individual ability to understand what other people think, and impute desires, intentions, and beliefs to oneself and others (Decety and Jackson, 2004; Decety and Moriguchi, 2007; Young et al., 2007; Blair, 2008; De Waal, 2008). ToM is an essential parameter in normal social interactions such as cooperating with colleagues and family members, thinking about others in their absence and anticipating their emotional reactions. As such, ToM is not only a cognitive construct but also involves an affective dimension based on the empathetic appreciation of the listener's emotional state (Shamay-Tsoory et al., 2009). Not surprisingly, ToM is severely affected in a variety of psychiatric disorders including autism, schizophrenia, bipolar disorder, and attention-deficit/hyperactivity disorder (Corcoran et al., 1995; Kerr et al., 2003; Abikoff et al., 2004; Blair, 2005; Bora and Pantelis, 2016; Maoz et al., 2019). Recent insights proposed that in neurotypical individuals, there are two ToM systems: one controlled that acts when we deliberately considers other's thoughts and emotions and the other implicit that involves the automatic analysis of their viewpoints even when such analysis is irrelevant for task processing (Onishi and Baillargeon, 2005; Surian et al., 2007; Kovacs et al., 2010; Schneider et al., 2012). Implicit ToM is thought to be developed early during development and remain stable over life span (Schneider et al., 2012). Although there is a wide agreement that adult humans are able to engage in unconscious analyses of others' mental states (Schneider et al., 2017), several studies led to conflicting data regarding the reliability of the implicit ToM using non-verbal measures such as violation of expectation paradigms and interactive and anticipatory looking tasks (for review, see Kulke and Hinrichs, 2021).

Automatic perspective taking (APT) seems to be the most reliable facet of implicit ToM. Using the dot-perspective-taking task developed by Samson et al. (2010, Experiment 1), previous studies demonstrated that people automatically take into account others' perspective even when it prevents them from achieving their own goals (Qureshi and Monk, 2018; Qureshi et al., 2020). Although there is an ongoing theoretical debate of whether automatic interference effects in the dot-perspective task are the product of domain-specific perspective-taking processes or of domain-general submentalizing processes (for review, see Cole and Millett, 2019 and Westra et al., 2021), recent lines of evidence suggest that this human ability may be of key importance for the ascription of mental states and social interactions (Furlanetto et al., 2016; Gao et al., 2019). In particular, Drayton et al. (2018) reported that psychopaths are able to represent others' perspective in goal-conducive tasks but show a striking ability of ignoring it in non-goal-relevant situations. They postulated that their lack of the ability to automatically represent the belief states of others when it does not serve their own ends may be at the origin of their maladaptive social behavior. However, this study included only offenders from a high-security hospital without control groups.

Automatic perspective taking is a complex phenomenon that may be impacted by attention and global intelligence as well as levels of social cognition (Schneider et al., 2012; Burnside et al., 2017; Pineda-Alhucema et al., 2018). To date, there is no study addressing the cognitive and emotional determinants of APT in healthy adults. Our hypothesis is that APT is independent of explicit ToM as well as affective empathy performance but may be affected by impulsivity and attention as well as low levels of global intelligence. To address this issue, we examined the performance in the Samson et al. APT task in 91 healthy adults who underwent a detailed neuropsychological testing including assessment of their general intelligence, attention and impulsivity, alexithymia, and measures of affective empathy and explicit ToM.

## Materials and Methods

### Participants

The study was approved by the local Ethics Committee and all participants gave written informed consent prior to inclusion. The present sample included 91 community-dwelling young men (mean age: 32.9, age range: 19–66) recruited via advertisements in local newspapers and social media. The following exclusion criteria were applied: (a) presence or history of a chronic psychiatric disorder (psychosis or bipolar disorder), (b) history of loss of consciousness lasting longer than 30 min, (c) history of head injury or post-concussion symptoms, (d) history of auditory or visual deficits, seizure, and neurological disorders, and (e) regular use of psychotropic medications.

### Neuropsychological Assessment

The Wechsler Adult Intelligence Scale (WAIS) is a general intelligence battery (Wechsler, 2011) used to evaluate patient's intelligence quotient (IQ). The ten core subtests of the battery give rise to four index scores including the Verbal Comprehension Index, the Perceptual Reasoning Index, the Working Memory Index, and the Processing Speed Index.

The Conners' Continuous Performance Test-II (CPT-II) is a computerized measure of inattentiveness, impulsivity, sustained attention, and vigilance (Conners, 2004). CPT-II outcome variables include hit reaction time to correct responses (Hit RT), standard error of HRT (Hit RT SE), omission errors (missed targets), commission errors (incorrect responses to non-targets), and detectability (ability to discriminate between targets and non-targets).

The French version of the Toronto Alexithymia Scale (TAS; Pinaquy et al., 2002) is a 20-item instrument that is one of the most commonly used measures of alexithymia. Alexithymia refers to people who have trouble identifying and describing emotions and who tend to minimize emotional experience and focus attention externally. Items are rated using a 5-point Likert scale whereby 1 = strongly disagree and 5 = strongly agree. There are 5 items that are negatively keyed (items 4, 5, 10, 18, and 19). The total alexithymia score is the sum of responses to all 20 items. The TAS-20 uses cutoff scoring: ≤51 = non-alexithymia; ≥61 = alexithymia; scores of 52–60 = possible alexithymia.

The Geneva Social Cognition Scale (GeSoCS) is a medium duration assessment tool that detects and characterizes significant changes in social cognition and ToM. It is a 100-point scale composed of six subtests: ToM stories, recognition of social emotions, false beliefs, inferences, absurdity judgment, and planning abilities (Martory et al., 2015).

The mini-Social cognition and Emotional Assessment (SEA) is a quick clinical tool that assesses emotion recognition and ToM deficits. One part is a reduced and modified version of the faux-pas test, and the second part is a reduced version of Paul Ekman emotion recognition test (Bertoux et al., 2014).

### Visual Perspective-Taking Task

We used the computer-based response-time task developed by Samson and colleagues (Samson et al., 2010). The stimuli consisted of a picture showing a lateral view into a room with the left, back, and right walls visible, and with red disks displayed on one or two walls. A human avatar always appeared in the center of the room facing either the right or the left wall. Depending upon the orientation of the avatar and the positioning of the disks, the avatar was able or unable to see all of the disks in the room. On each trial, participants judged either their own visual perspective (self-trials) or the visual perspective of the avatar (avatar trials). Specifically, participants were asked to verify the number of disks that either they (self) or the avatar could see. On 50% trials, the participant and the avatar could see the same number of disks (consistent perspective condition). On 50% trials, they could see different numbers of disks (inconsistent perspective condition). The position of the avatar was kept constant across consistent and inconsistent trials, but the position of the disks changed. Each trial included four stimuli, presented in the center of the screen in the following order: (i) a fixation cross indicating the start of the trial, (ii) a word indicating whether participants should adopt their own perspective (“YOU”) or the perspective of the avatar (“HE”), (iii) a number (0–3) specifying the content to be verified, and (iv) a picture of the avatar in a room. Stimuli i–iii each appeared for 750 ms, and each one was followed by a blank screen for 500 ms. After the final stimulus, participants had 2,000 ms to indicate whether the picture matched the specified perspective and content (“yes” response), or that it did not match the specified perspective and content (“no” response). In case of “no” answer (2,000 ms), the next trial became available. Participants did not receive any trial-by-trial feedback about their performance. Trials were presented in four blocks, each consisting of 48 trials. Each block also included four filler trials in which there were no disks on the walls of the room. These filler trials were included to ensure that the correct response to the perspective “YOU” and content “0” were sometimes “yes”. The order of presentation of the blocks were randomized and counterbalanced across participants. Before beginning the experimental trials, each participant completed 26 practice trials. The entire procedure was conducted using DMDX software (developed by the University of Arizona) to control the stimulus presentation and data collection (Forster and Forster, 2003).

Anticipatory responses (<200 ms) or delayed responses (>2,000 ms) were counted as errors. Moreover, individuals with mean correct response reaction time over three 3SD from the participant's mean values were considered outliers and excluded from further analysis. The response times were log-transformed to normalize their distribution. Mean number of errors and reaction times were assessed for each of the four trial types (Self-Consistent, Self-Inconsistent, Other-Consistent, and Other-Inconsistent). Erroneous responses on all conditions (Self, Other, Consistent, and Inconsistent) were considered to define the mean number of errors in all of the cases. We also examined the determinants of egocentric vs. altercentric interference in this test. Egocentric interference corresponds to slower mean reaction time and more errors in the inconsistent compared to the consistent conditions when participants judged the avatars perspective. Altercentric interference refers to slower mean reaction time and more errors in the inconsistent compared to the consistent conditions when participants judge their own perspective. Egocentric interference was calculated by subtracting each participant's mean response time and number of errors on Other-Consistent from those on Other-Inconsistent trials. Altercentric interference was measured by subtracting each participant's mean response time and number of errors on Self-Consistent from those on Self-Inconsistent trials.

### Statistical Analysis

Multiple linear regression models (adjusted for age) were used to explore the association between mean reaction times (respectively, egocentric and altercentric interference in mean number of errors) in the visual perspective-taking task (dependent variables) and CPT-II parameters, WAIS global score (as well as the score of subscales), TAS, and GeSoCS and mini-SEA scores (independent variables). The association of the same neuropsychological tests (independent variables) with the sum of the number of errors across different conditions (self or other) were explored with multiple negative binomial regression adjusted for age. Correction for multiple analysis was made using the Benjamini–Hochberg method. We applied the Benjamini–Hochberg correction in three different sets of variables as a function of *a-priori* hypotheses: general intelligence (WAIS), attentional resources (CPT-II), and markers of social cognition and emotional regulation (TAS-20, GeSoCS, and mini-SEA). The corrected values of *p* took into account all of the comparisons made for each set of variables in Self and Other conditions (Green and Diggle, 2007).

## Results

The descriptive data of the present sample are summarized in Table 1. Among the independent variables included in the regression models, only the CPT-II parameters were significantly associated with the mean reaction times after Benjamini–Hochberg correction for multiple comparisons.

**Table 1:** Participant's characteristics (*N* = 91).

**Variable**	**Mean ±SD, 95% CI or *N* (%)**
Age	32.9 ± 10.1, 19.0–66.0
Education
<12 y	1 (1.1%)
12 y	23 (25.3%)
>12 y	67 (73.6%)
Right-handedness	86 (94.5%)
Neuropsychological tests
WAIS
Verbal comprehension index	37.7 ± 8.3, 20.0–54.0
Perceptual reasoning index	31.3 ± 7.2, 16.0–46.0
Working memory index	22.9 ± 4.9, 9.0–34.0
Processing speed index	20.0 ± 4.2, 9.0–33.0
IQ total score	111.9 ± 19.3, 67.0–152.0
General ability index (GAI)	69.0 ± 13.8, 42.0–97.0
CPT-II
Omission errors	50.0 ± 13.3, 39.9–122.9
Commission errors	49.6 ± 9.1, 32.9–74.0
Detectability	48.8 ± 8.7, 23.4–65.0
Hit reaction time (HRT)	47.3 ± 9.4, 25.5–73.5
HRT-SE	48.3 ± 10.4, 25.0–81.1
TAS Total score	40.6 ± 10.4, 0.0–67.0
GeSoCS Total Score	90.0 ± 5.8, 72.0–98.0
mini-SEA	26.7 ± 2.1, 20.6–30.0
Visual perspective-taking task
Mean reaction time (ms)	6,466.1 ± 1,489.8, 3,480.4–10,001.8
Self condition (ms)	816.9 ± 196.1, 434.8–1,334.1
Other condition (ms)	799.6 ± 186.1, 429.4–1,245.1
Egocentric interference (other)	−96.9 ± 64.9, −257.2 to 36.0
Altercentric interference (self)	−51.6 ± 126.4, −243.3 to 961.9
Number of errors
Sum of self	2.6 ± 3.5, 0.0–17.0
Sum of other	2.5 ± 2.7, 0.0–18.0
Egocentric interference (other)	−0.6 ± 1.0, −4.5 to 1.5
Altercentric interference (self)	−0.8 ± 1.3, −6.0 to 1.0

CPT-II HRT and hit RT SE (two measures of inattentiveness and impulsivity) were positively associated with the mean reaction time in the Self condition [regression coefficient 5.58 (95% CI: 1.52, 9.63) and 5.33 (95% CI: 1.78, 8.87), respectively]. The mean reaction time in the Other condition was positively related to CPT-II HRT [regression coefficient 4.71 (95% CI: 1.40, 8.02)] (Table 2).

**Table 2:** Association of neuropsychological tests (independent variables) with mean reaction time using linear regressions, adjusted for age.

	**Mean reaction time (self condition)**					**Mean reaction time (other condition)**				
	**Age**		**Neuro psy**			**Age**		**Neuro psy**		
**Neuro psy tests**	**Coeff (95% CI)**	***P***	**Coeff (95% CI)**	***P***	***P*** **_BH_**	**Coeff (95% CI)**	***P***	**Coeff (95% CI)**	***P***	***P*** **_BH_**
WAIS
Verbal comprehension index	8.63 (4.93, 12.33)	<0.001	−4.59 (−9.11, −0.08)	0.046	0.004	8.85 (5.39, 12.30)	<0.001	−3.86 (−8.08, 0.36)	0.072	0.004
Perceptual reasoning index	8.82 (5.07, 12.57)	<0.001	−5.20 (−10.49, 0.10)	0.054	0.004	9.27 (5.82, 12.71)	<0.001	−5.99 (−10.85, −1.12)	0.016	0.004
Working memory index	8.05 (4.35, 11.76)	<0.001	−4.12 (−11.85, 3.60)	0.292	0.004	8.37 (4.92, 11.81)	<0.001	−3.56 (−10.75, 3.62)	0.327	0.004
Processing speed index	8.25 (4.71, 11.79)	<0.001	−13.30 (−21.79, −4.81)	0.005	0.004	8.55 (5.26, 11.84)	<0.001	−12.34 (−20.23, −4.45)	0.005	0.004
IQ total score	8.82 (5.16, 12.47)	<0.001	−2.48 (−4.40, −0.56)	0.012	0.004	9.10 (5.71, 12.49)	<0.001	−2.37 (−4.15, −0.59)	0.010	0.004
IAG	8.91 (5.19, 12.63)	<0.001	−3.08 (−5.80, −0.35)	0.027	0.004	9.21 (5.77, 12.66)	<0.001	−3.02 (−5.54, −0.50)	0.020	0.004
CPT-II
Omission errors	7.90 (4.17, 11.63)	<0.001	0.85 (−1.99, 3.70)	0.552	0.010	8.31 (4.84, 11.79)	<0.001	−0.05 (−2.70, 2.60)	0.970	0.010
Commission errors	7.93 (4.20, 11.65)	<0.001	−1.12 (−5.26, 3.02)	0.592	0.010	8.29 (4.82, 11.76)	<0.001	−0.36 (−4.21, 3.49)	0.853	0.010
Detectability	8.00 (4.27, 11.73)	<0.001	0.82 (−3.50, 5.15)	0.705	0.010	8.34 (4.88, 11.79)	<0.001	1.51 (−2.50, 5.52)	0.455	0.010
Hit reaction time (HRT)	6.46 (2.71, 10.21)	0.001	5.58 (1.52, 9.63)	**0.008**	0.010	7.19 (3.65, 10.73)	<0.001	4.08 (0.26, 7.91)	0.037	0.010
HRT-SE	6.93 (3.31, 10.55)	<0.001	5.33 (1.78, 8.87)	**0.004**	0.010	7.37 (3.99, 10.76)	<0.001	4.71 (1.40, 8.02)	**0.006**	0.010
TAS Total score	7.99 (4.31, 11.68)	<0.001	2.63 (−0.98, 6.24)	0.151	0.004	8.31 (4.85, 11.77)	<0.001	1.27 (−2.12, 4.65)	0.459	0.004
GeSoCS Total Score	7.93 (4.19, 11.67)	<0.001	−1.20 (−7.79, 5.39)	0.719	0.004	8.28 (4.81, 11.76)	<0.001	−0.56 (−6.69, 5.57)	0.856	0.004
mini-SEA	7.98 (4.26, 11.70)	<0.001	−5.97 (−23.90, 11.96)	0.510	0.004	8.30 (4.84, 11.75)	<0.001	−6.89 (−23.53, 9.76)	0.413	0.004

*Benjamini–Hochberg threshold, P_BH_; CPT-II, Conners' Continuous Performance Test-II; TAS, French version of the Toronto Alexithymia Scale; GeSoCS, Geneva Social Cognition Scale; mini-SEA, mini-Social cognition and Emotional Assessment. Values in bold correspond to significant results after correction for multiple comparisons*.

The WAIS global score was negatively associated with mean reaction time in the Self condition [regression coefficient for the Self condition: −2.48 (95% CI: −4.40, −0.56)]. In the Other condition, this association was also statistically significant [regression coefficient for Other condition −2.37 (95% CI: −4.15, −0.59)]. Similar data were obtained for some of the WAIS subscales. However, these associations did not persist after correction for multiple comparisons (Table 2).

The increased number of commissions as well as increased detectability and HRT-SE in CPT-II were all related to increased number of errors in Self condition (regression coefficients from 0.03 to 0.04). CPT-II HRT-SE was also related to worse performances in Other condition (regression coefficient 0.02) (Table 3).

**Table 3:** Association of neuropsychological tests (independent variables) with the sum of the number of errors across different conditions (self or other) using negative binomial regressions, adjusted for age.

	**Number of errors (sum of self)**					**Number of errors (sum of other)**				
	**Age**		**Neuropsy**			**Age**		**Neuropsy**		
**Neuropsy tests**	**IRR (95% CI)**	***P***	**IRR (95% CI)**	***P***	***P*** **_BH_**	**IRR (95% CI)**	***P***	**IRR (95% CI)**	***P***	***P*** **_BH_**
WAIS
Verbal comprehension index	−0.02 (−0.04, 0.00)	0.109	0.00 (−0.03, 0.03)	0.994	0.004	−0.02 (−0.04, 0.00)	0.055	−0.00 (−0.03, 0.02)	0.825	0.004
Perceptual reasoning index	−0.02 (−0.04, 0.01)	0.180	−0.03 (−0.07, 0.01)	0.121	0.004	−0.02 (−0.04, 0.00)	0.089	−0.02 (−0.05, 0.01)	0.193	0.004
Working memory index	−0.02 (−0.04, 0.00)	0.116	0.01 (−0.05, 0.06)	0.845	0.004	−0.02 (−0.04, 0.00)	0.055	0.00 (−0.04, 0.05)	0.879	0.004
Processing speed index	−0.02 (−0.04, 0.00)	0.108	0.04 (−0.03, 0.11)	0.292	0.004	−0.02 (−0.04, 0.00)	0.051	0.03 (−0.02, 0.08)	0.217	0.004
IQ total score	−0.02 (−0.04, 0.00)	0.111	−0.00 (−0.02, 0.01)	0.750	0.004	−0.02 (−0.04, 0.00)	0.056	−0.00 (−0.01, 0.01)	0.786	0.004
General ability index (GAI)	−0.02 (−0.04, 0.01)	0.124	−0.01 (−0.03, 0.01)	0.449	0.004	−0.02 (−0.04, 0.00)	0.068	−0.01 (−0.02, 0.01)	0.433	0.004
CPT-II
Omission errors	−0.02 (−0.05, 0.00)	0.087	0.01 (−0.01, 0.02)	0.363	0.020	−0.02 (−0.04, 0.00)	0.053	0.00 (−0.01, 0.01)	0.856	0.020
Commission errors	−0.02 (−0.04, 0.01)	0.125	0.04 (0.01, 0.07)	**0.004**	0.020	−0.02 (−0.04, 0.00)	0.087	0.02 (−0.01, 0.04)	0.140	0.020
Detectability	−0.02 (−0.04, 0.00)	0.085	0.04 (0.01, 0.08)	**0.009**	0.020	−0.02 (−0.04, 0.00)	0.071	0.02 (−0.01, 0.04)	0.217	0.020
Hit reaction time (HRT)	−0.03 (−0.05, 0.00)	0.046	0.02 (−0.01, 0.05)	0.148	0.020	−0.03 (−0.05, −0.01)	0.013	0.03 (0.00, 0.05)	0.034	0.020
HRT-SE	−0.02 (−0.05, 0.00)	0.056	0.03 (0.01, 0.05)	**0.008**	0.020	−0.02 (−0.04, −0.00)	0.035	0.02 (0.00, 0.04)	**0.014**	0.020
TAS Total score	−0.02 (−0.05, 0.00)	0.072	0.01 (−0.01, 0.03)	0.339	0.008	−0.02 (−0.04, −0.00)	0.047	0.01 (−0.00, 0.03)	0.114	0.008
GeSoCS Total Score	−0.03 (−0.05, 0.01)	0.017	−0.07 (−0.12, −0.03)	**0.002**	0.008	−0.02 (−0.04, −0.00)	0.022	−0.03 (−0.06, 0.01)	0.108	0.008
mini-SEA	−0.02 (−0.05, 0.00)	0.065	−0.05 (−0.19, 0.09)	0.503	0.008	−0.02 (−0.04, −0.00)	0.027	−0.04 (−0.14, 0.06)	0.448	0.008

*Benjamini–Hochberg threshold, P_BH_; WAIS, Wechsler Adult Intelligence Scale; CPT-II, Conners' Continuous Performance Test-II; TAS, French version of the Toronto Alexithymia Scale; GeSoCS, Geneva Social Cognition Scale; mini-SEA: mini-Social cognition and Emotional Assessment. Values in bold correspond to significant results after correction for multiple comparisons*.

The GeSoCS score was negatively associated with the number of errors in Self condition [regression coefficient: −0.07 (95% CI: −0.12, −0.03)] (Table 3).

Among the variables studied, only the CPT-II parameters have a significant impact on egocentric and altercentric interference. Egocentric interference for the number of errors was negatively related to CPT-II HRT-SE [regression coefficient: −0.03 (95% CI: 0.05, 0.01), adjusted *p* = 0.0034]. Altercentric interference for the number of errors was negatively related to CPT-II Omissions [regression coefficient: −0.04 (95% CI: 0.05, 0.02), adjusted *p* = 0.0002], Commissions [regression coefficient: −0.04 (95% CI: 0.07, 0.01), adjusted *p* = 0.0086], and HRT-SE [regression coefficient: −0.04 (95% CI: 0.07, 0.02), *p* = 0.0005; Table 4]. No significant association was found between CPT-II variables and both egocentric and altercentric interference for the mean reaction times.

**Table 4:** Association of neuropsychological tests (independent variables) with egocentric and altercentric interferences using linear regressions, adjusted for age.

	**Altercentric interference in number of errors (self)**				**Egocentric interference in number of errors (other)**			
	**Age**		**Neuropsy**		**Age**		**Neuropsy**	
**Neuro psy tests**	**Coeff (95% CI)**	***P***	**Coeff (95% CI)**	***P***	**Coeff (95% CI)**	***P***	**Coeff (95% CI)**	***P***
**CPT-II**
Omission errors	0.02 (−0.00, 0.05)	0.0823	−0.04 (−0.05, −0.02)	**0.0002**	0.02 (0.00, 0.04)	0.0321	−0.00 (−0.02, 0.01)	0.7325
Commission errors	0.02 (−0.01, 0.04)	0.2162	−0.04 (−0.07, −0.01)	**0.0086**	0.02 (0.00, 0.04)	0.0347	−0.00 (−0.02, 0.02)	0.8952
Detectability	0.02 (−0.01, 0.04)	0.1831	−0.03 (−0.06, −0.00)	0.0281	0.02 (0.00, 0.04)	0.0335	0.00 (−0.02, 0.02)	0.8776
Hit reaction time (HRT)	0.02 (−0.01, 0.05)	0.1268	−0.01 (−0.04, 0.02)	0.4122	0.03 (0.01, 0.05)	0.0072	−0.02 (−0.05, −0.00)	0.0337
HRT-SE	0.03 (0.00, 0.05)	0.0369	−0.04 (−0.07, −0.02)	**0.0005**	0.03 (0.01, 0.05)	0.0061	−0.03 (−0.05, −0.01)	**0.0034**

*Benjamini–Hochberg threshold, P_BH_ = 0.020; CPT-II, Conners' Continuous Performance Test-II. Values in bold correspond to significant results after correction for multiple comparisons*.

## Discussion

Our data show that CPT-II parameters of attention and impulsivity have a direct and strong impact on APT performances assessed with the dot-perspective task in healthy adults. Attention and impulsivity levels are associated with both mean reaction times and mean number of errors in the current experimental paradigm. Although intuitively evident, the impact of attention and impulsivity is quite different in Self compared to Other conditions. When participants focus on their own perspective, their mean reaction time increases as a function of the two measures of inattentiveness and impulsivity, namely, the hit RT, a measure of response time, and HRT-SE, a measure of the consistency of response time in CPT-II. In contrast, when participants report the avatar's view, their mean reaction time decreases in cases with higher number of omissions (that assess the level of inattentiveness). In other words, people with decreased levels of attention and higher impulsivity may need more time to define their own perspective (both in consistent and inconsistent trials) but tend to precipitate their response when they should take the perspective of others. Most importantly, CPT-II parameters assessing inattentiveness and impulsivity are associated with increased number of errors both in Self and Other conditions. Of importance, these parameters were also negatively related to egocentric and altercentric interference at the level of mean number of errors but not mean reaction time. Gardner et al. (2018) reported that attentional orienting contributes decisively to performance in the dot-perspective task (Gardner et al., 2018). More recently, O'Grady et al. (2020) postulated that APT is a rapid, unconscious, and involuntary phenomenon, referred to as spontaneous but not automatic, that needs preserved attention. The present findings parallel these observations, suggesting that people with lower attentional resources display worse performances in terms of number of errors, are less able to manage their reaction time both in Self and Other conditions, and are less responsive to both egocentric and altercentric interferences. A very recent study by Qureshi et al. (2020), using the same experimental paradigm, demonstrated that conflict indices (consistent-inconsistent perspective) are related to stop-signal inhibitory control task (but not go–no-go or shape-matching tasks) and indicate that this selection may occur very late in cognitive processing. In contrast, altercentric interference was not related to any of the executive tasks in this study. Taking together, these data indicate that attentional resources rather than inhibitory control abilities may impact on self–other interference when performing a visual perspective task. Pointing to the importance of adjusting for global empathy levels, the mean number of errors in our cohort was negatively associated with the GeSoCS score, a composite measure of explicit ToM and affective empathy. However, this global empathy score was not related to mean reaction times and did not impact on egocentric and altercentric interference. Neither global intelligence nor alexithymia have an effect on dot-perspective task performances. This observation agrees with several reports showing that dot-perspective task performances may be preserved in psychiatric pathologies affecting intelligence but also emotional regulation such as autism spectrum conditions and alcoholism (Pearson et al., 2013; Cox et al., 2016, 2018; Sijtsma et al., 2020).

To our knowledge, this is the first study that analyzes the cognitive and emotional determinants of APT performance in community-dwelling young adults. Our results indicate that APT mostly depends on attentional resources and impulsivity even in healthy adults. It is likely that patients with specific alterations of attention and impulsive control, such as patients with attention deficit and hyperactivity disorder (ADHD) and borderline personality, may fail to activate this human ability. The strengths of this report include its use of a large sample, independent variables assessing cognitive processes such as attention and fluid intelligence, general levels of empathy, but also emotional regulation (alexithymia, impulsivity) and stringent control for multiple comparisons across the regression models. Some limitations should also be noted. All of our cases were socially integrated young individuals without history of criminal convictions and substance abuse. The careful exclusion of neurological and psychiatric disorders as well as regular use of psychotropics and CPT-II performances within the normal range limits the generalizability of our observations. Moreover, no direct assessment of inhibitory control and cognitive flexibility was performed as part of the neuropsychological analysis. These parameters are known to impact performances on visual perspective taking (Qureshi and Monk, 2018; Qureshi et al., 2020; Li et al., 2021). Future studies in mixed series including patients with ADHD and borderline personality, investigation of social parameters (job loss, separation), and careful analysis of executive functions are needed to obtain a better insight into the social relevance of APT concept.

## Data Availability Statement

The raw data supporting the conclusions of this article will be made available by the authors, without undue reservation.

## Ethics Statement

The studies involving human participants were reviewed and approved by Commission cantonale d'éthique de la recherche (CCER). The patients/participants provided their written informed consent to participate in this study.

## Author Contributions

PG, AP, and M-LM: conceived the study. M-LM: conceived the visual perspective-taking task. CR and M-LM: recruitment. M-LM, CR, and PG: neuropsychology supervising. M-LM and FH: data preparation. FH, PG, and M-LM: analyzed the data. PG, AP, FH, and M-LM: manuscript writing. All authors contributed to the article and approved the submitted version.

## Funding

This research was funded by the Swiss National Foundation (Grant number 320030_189247).

## Conflict of Interest

The authors declare that the research was conducted in the absence of any commercial or financial relationships that could be construed as a potential conflict of interest.

## Publisher's Note

All claims expressed in this article are solely those of the authors and do not necessarily represent those of their affiliated organizations, or those of the publisher, the editors and the reviewers. Any product that may be evaluated in this article, or claim that may be made by its manufacturer, is not guaranteed or endorsed by the publisher.

## References

[B1] Abikoff H. Hechtman L. Klein R. G. Gallagher R. Fleiss K. Etcovitch J.. (2004). Social functioning in children with ADHD treated with long-term methylphenidate and multimodal psychosocial treatment. J. Am. Acad. Child Adolesc. Psychiatry 43, 820–829. 10.1097/01.chi.0000128797.91601.1a 15213583

[B2] Bertoux M. Volle E. De Souza L. C. Funkiewiez A. Dubois B. Habert M. O. (2014). Neural correlates of the mini-SEA (Social cognition and Emotional Assessment) in behavioral variant frontotemporal dementia. Brain Imaging Behav. 8, 1–6. 10.1007/s11682-013-9261-0 24078043

[B3] Blair R. J. (2005). Responding to the emotions of others: dissociating forms of empathy through the study of typical and psychiatric populations. Conscious. Cogn. 14, 698–718. 10.1016/j.concog.2005.06.004 16157488

[B4] Blair R. J. R. (2008). Fine cuts of empathy and the amygdala: dissociable deficits in psychopathy and autism. Q. J. Exp. Psychol. 61, 157–170. 10.1080/17470210701508855 18038346

[B5] Bora E. Pantelis C. (2016). Meta-analysis of social cognition in attention-deficit/hyperactivity disorder (ADHD): comparison with healthy adults and autistic spectrum disorder. Psychol. Med. 46, 699–716. 10.1017/S0033291715002573 26707895

[B6] Burnside K. Wright K. Poulin-Dubois D. (2017). Social motivation and implicit theory of mind in children with autism spectrum disorder. Autism Res. 10, 1834–1844. 10.1002/aur.1836 28762662 PMC5772680

[B7] Cole G. G. Millett A. C. (2019). The closing of the theory of mind: a critique of perspective-taking. Psychon. Bull. Rev. 26, 1787–1802. 10.3758/s13423-019-01657-y 31515733

[B8] Conners C. K. (2004). Conners' Continuous Performance Test. 2nd Edn. Toronto, ON: Multi-Health Systems.

[B9] Corcoran R. Mercer G. Frith C. D. (1995). Schizophrenia, symptomatology and social inference: investigating “theory of mind” in people with schizophrenia. Schizophr. Res. 17, 5–13. 10.1016/0920-9964(95)00024-G 8541250

[B10] Cox S. Chandler C. Simpson A. Riggs K. (2016). The effect of alcohol dependence on automatic visuo-spatial perspective taking. Drug Alcohol Depend. 166, 21–25. 10.1016/j.drugalcdep.2016.06.007 27350654

[B11] Cox S. Maurage P. O'connor R. Chandler C. Riggs K. (2018). Automatic visual-spatial perspective taking in alcohol-dependence: a study with happy emotional faces. Drug Alcohol Depend. 190, 42–45. 10.1016/j.drugalcdep.2018.05.025 29966852

[B12] De Waal F. B. M. (2008). Putting the altruism back into altruism: the evolution of empathy. Annu. Rev. Psychol. 59, 279–300. 10.1146/annurev.psych.59.103006.093625 17550343

[B13] Decety J. Jackson P. L. (2004). The functional architecture of human empathy. Behav. Cogn. Neurosci. Rev. 3, 71–100. 10.1177/1534582304267187 15537986

[B14] Decety J. Moriguchi Y. (2007). The empathic brain and its dysfunction in psychiatric populations: implications for intervention across different clinical conditions. Biopsychosoc. Med. 1, 22. 10.1186/1751-0759-1-22 18021398 PMC2206036

[B15] Drayton L. A. Santos L. R. Baskin-Sommers A. (2018). Psychopaths fail to automatically take the perspective of others. Proc. Natl. Acad. Sci. U. S. A. 115, 3302–3307. 10.1073/pnas.1721903115 29531085 PMC5879707

[B16] Forster K. I. Forster J. C. (2003). DMDX: a windows display program with millisecond accuracy. Behav. Res. Methods Instrum. Comput. 35, 116–124. 10.3758/BF03195503 12723786

[B17] Furlanetto T. Becchio C. Samson D. Apperly I. (2016). Altercentric interference in level 1 visual perspective taking reflects the ascription of mental states, not submentalizing. J. Exp. Psychol. Hum. Percept. Perform. 42, 158–163. 10.1037/xhp0000138 26389611

[B18] Gao Q. Chen W. Wang Z. Lin D. (2019). Secret of the masters: young chess players show advanced visual perspective taking. Front. Psychol. 10, 2407. 10.3389/fpsyg.2019.02407 31708844 PMC6821682

[B19] Gardner M. R. Hull Z. Taylor D. Edmonds C. J. (2018). 'Spontaneous' visual perspective-taking mediated by attention orienting that is voluntary and not reflexive. Q. J. Exp. Psychol. 71, 1020–1029. 10.1080/17470218.2017.1307868 28303749

[B20] Green G. H. Diggle P. J. (2007). On the operational characteristics of the Benjamini and Hochberg False Discovery Rate procedure. Stat. Appl. Genet. Mol. Biol. 6, 27. 10.2202/1544-6115.1302 18052910

[B21] Kerr N. Dunbar R. I. Bentall R. P. (2003). Theory of mind deficits in bipolar affective disorder. J. Affect. Disord. 73, 253–259. 10.1016/S0165-0327(02)00008-3 12547294

[B22] Kovacs A. M. Teglas E. Endress A. D. (2010). The social sense: susceptibility to others' beliefs in human infants and adults. Science 330, 1830–1834. 10.1126/science.1190792 21205671

[B23] Kulke L. Hinrichs M. A. B. (2021). Implicit Theory of Mind under realistic social circumstances measured with mobile eye-tracking. Sci. Rep. 11, 1215. 10.1038/s41598-020-80614-5 33441890 PMC7806733

[B24] Li X. Yuan M. Xu P. Wu W. (2021). Inhibitory control was needed in level-1 visual perspective taking: a developing negative priming study. Psychol. Res. Behav. Manag. 14, 1779–1788. 10.2147/PRBM.S333824 34764705 PMC8572879

[B25] Maoz H. Gvirts H. Z. Sheffer M. Bloch Y. (2019). Theory of mind and empathy in children with ADHD. J. Atten. Disord. 23, 1331–1338. 10.1177/1087054717710766 28558473

[B26] Martory M. D. Pegna A. J. Sheybani L. Metral M. Bernasconi Pertusio F. Annoni J. M. (2015). Assessment of social cognition and theory of mind: initial validation of the Geneva social cognition scale. Eur. Neurol. 74, 288–295. 10.1159/000442412 26656509

[B27] O'Grady C. Scott-Phillips T. Lavelle S. Smith K. (2020). Perspective-taking is spontaneous but not automatic. Q. J. Exp. Psychol. 73, 1605–1628. 10.1177/1747021820942479 32718242 PMC7551223

[B28] Onishi K. H. Baillargeon R. (2005). Do 15-month-old infants understand false beliefs? Science 308, 255–258. 10.1126/science.1107621 15821091 PMC3357322

[B29] Pearson A. Ropar D. De C. H. a.F. (2013). A review of visual perspective taking in autism spectrum disorder. Front. Hum. Neurosci. 7, 652. 10.3389/fnhum.2013.00652 24115930 PMC3792367

[B30] Pinaquy S. Chabrol H. Barbe P. (2002). [Factorial analysis and internal consistency of the French version of the Toronto Alexithymia Scale (TAS 20), in obese women]. Encephale 28, 277–282. 12232536

[B31] Pineda-Alhucema W. Aristizabal E. Escudero-Cabarcas J. Acosta-Lopez J. E. Velez J. I. (2018). Executive function and theory of mind in children with ADHD: a systematic review. Neuropsychol. Rev. 28, 341–358. 10.1007/s11065-018-9381-9 30168020

[B32] Qureshi A. W. Monk R. L. (2018). Executive function underlies both perspective selection and calculation in Level-1 visual perspective taking. Psychon. Bull. Rev. 25, 1526–1534. 10.3758/s13423-018-1496-8 29949017 PMC6096641

[B33] Qureshi A. W. Monk R. L. Samson D. Apperly I. A. (2020). Does interference between self and other perspectives in theory of mind tasks reflect a common underlying process? Evidence from individual differences in theory of mind and inhibitory control. Psychon. Bull. Rev. 27, 178–190. 10.3758/s13423-019-01656-z 31429057 PMC7000534

[B34] Samson D. Apperly I. A. Braithwaite J. J. Andrews B. J. Bodley Scott S. E. (2010). Seeing it their way: evidence for rapid and involuntary computation of what other people see. J. Exp. Psychol. Hum. Percept. Perform. 36, 1255–1266. 10.1037/a0018729 20731512

[B35] Schneider D. Lam R. Bayliss A. P. Dux P. E. (2012). Cognitive load disrupts implicit theory-of-mind processing. Psychol. Sci. 23, 842–847. 10.1177/0956797612439070 22760885

[B36] Schneider D. Slaughter V. P. Dux P. E. (2017). Current evidence for automatic Theory of Mind processing in adults. Cognition 162, 27–31. 10.1016/j.cognition.2017.01.018 28189035

[B37] Shamay-Tsoory S. G. Aharon-Peretz J. Perry D. (2009). Two systems for empathy: a double dissociation between emotional and cognitive empathy in inferior frontal gyrus versus ventromedial prefrontal lesions. Brain 132, 617–627. 10.1093/brain/awn279 18971202

[B38] Sijtsma H. Lee N. C. Hollarek M. Walsh R. J. Van Buuren M. Braams B. R.. (2020). Social cognition and friendships in adolescents with autistic-like experiences and psychotic-like experiences. Front. Psychiatry 11, 589824. 10.3389/fpsyt.2020.589824 33519546 PMC7843702

[B39] Surian L. Caldi S. Sperber D. (2007). Attribution of beliefs by 13-month-old infants. Psychol. Sci. 18, 580–586. 10.1111/j.1467-9280.2007.01943.x 17614865

[B40] Wechsler D. (2011). WAIS-IV: Echelle d'Intelligence de Wechsler Pour Adultes: Manuel d'Interprétation. Paris: ECPA.

[B41] Westra E. Terrizzi B. F. Van Baal S. T. Beier J. S. Michael J. (2021). Beyond avatars and arrows: testing the mentalising and submentalising hypotheses with a novel entity paradigm. Q. J. Exp. Psychol. 74, 1709–1723. 10.1177/17470218211007388 33752520 PMC8392802

[B42] Young L. Cushman F. Hauser M. Saxe R. (2007). The neural basis of the interaction between theory of mind and moral judgment. Proc. Natl. Acad. Sci. U. S. A. 104, 8235–8240. 10.1073/pnas.0701408104 17485679 PMC1895935

